# Identification of suitable reference genes for the relative quantification of microRNAs in pleural effusion

**DOI:** 10.3892/ol.2014.2404

**Published:** 2014-08-01

**Authors:** HYE-SUK HAN, YEONG NANG JO, JIN YONG LEE, SONG-YI CHOI, YUSOOK JEONG, JIEUN YUN, OK-JUN LEE

**Affiliations:** 1 Department of Internal Medicine, College of Medicine, Chungbuk National University, Cheongju 361-763, Republic of Korea; 2 Bioevaluation Center, Korea Research Institute of Bioscience and Biotechnology, Cheongwon 363-883, Republic of Korea; 3 Public Health Medical Service, SMG-SNU Boramae Medical Center, Seoul 156-707, Republic of Korea; 4 Department of Pathology, College of Medicine, Chungbuk National University, Cheongju 361-763, Republic of Korea

**Keywords:** microRNAs, pleural effusion, reference

## Abstract

Circulating cell-free microRNAs (miRNAs) are potential biomarkers of cancer. Reverse transcription-quantitative polymerase chain reaction (RT-qPCR) is widely used in miRNA expression studies. The aim of this study was to identify suitable reference genes for RT-qPCR analyses of miRNA expression levels in pleural effusion. The expression levels of candidate reference miRNAs were investigated in 10 benign pleural effusion (BPE) and 10 lung adenocarcinoma-associated malignant pleural effusion (LA-MPE) samples using miRNA microarrays. The expression levels of candidate reference miRNAs, together with those of U6 small nuclear RNA (snRNA), RNU6B, RNU44 and RNU48 small RNAs, in 46 BPE and 45 LA-MPE samples were validated by RT-qPCR, and were analyzed using the NormFinder and BestKeeper algorithms. The impact of different normalization approaches on the detection of differential expression levels of miR-198 in BPE and LA-MPE samples was also assessed. As determined by the miRNA microarray data, five candidate reference miRNAs were identified. Following RT-qPCR validation, U6 snRNA, miR-192, miR-20a, miR-221, miR-222 and miR-16 were evaluated using the NormFinder and BestKeeper software programs. U6 snRNA and miR-192 were identified as single reference genes and the combination of these genes was preferred for the relative quantification of miRNA expression levels in pleural effusion. Normalization of miR-98 expression levels to those of U6 snRNA, miR-192 or a combination of these genes enabled the detection of a significant difference between BPE and LA-MPE samples. Therefore, U6 snRNA and miR-192 are recommended as reference genes for the relative quantification of miRNA expression levels in pleural effusion.

## Introduction

MicroRNAs (miRNAs) constitute a class of small non-coding RNAs 19–24 nucleotides in length that function as post-transcriptional regulators of gene expression (1). miRNAs exert important regulatory roles in the majority of cellular and developmental processes, and have been implicated in numerous human diseases, including cancer (2). Since their recent identification, the investigation of the potential for using extracellular and circulating miRNAs as non-invasive cancer biomarkers in body fluids, such as serum and plasma, has rapidly expanded (3,4). Circulating miRNAs are stabilized and protected from RNase degradation by incorporation into various protein complexes or membranous particles, including exosomes and microvesicles (4).

Pleural effusion is a common clinical manifestation of the various types of non-small cell lung cancer, particularly adenocarcinoma, and the diagnosis of pleural effusion in these patients is of particular clinical importance. According to the Cancer Staging system, the existence of malignant pleural effusion (MPE) at stage IV indicates systemic disease; these patients cannot be treated with local therapeutic methods, such as surgical resection or radiotherapy (5). Currently the diagnosis of MPE relies on cytological analysis of pleural fluid; however, this method has limited sensitivity (6) and alternative methods are required to improve the diagnosis of pleural effusion. Recently, several studies have reported that the cell-free miRNAs present in pleural effusion may be useful diagnostic biomarkers for discriminating between benign pleural effusion (BPE) and MPE (7–9).

miRNA expression levels have been analyzed using traditional semi-quantitative methods, including northern blotting, bead-based flow-cytometry and microarray technology; however, reverse transcription-quantitative polymerase chain reaction (RT-qPCR) is the most sensitive, reproducible and widely used approach for the evaluation of miRNAs (10). To prevent errors in datasets and to overcome the experimental variation associated with RT-qPCR analysis procedures, such as RNA isolation, cDNA synthesis and PCR kinetics, the relative quantification of miRNAs through normalization to one or more stably expressed reference gene is the preferred approach (10). Suitable reference genes should be expressed constitutively and the expression levels should be unaffected by biological change, disease or treatment. The normalization of experimental gene RT-qPCR data to those of unreliable reference genes may result in incorrect quantification of the miRNAs of interest; the importance of validating suitable candidate endogenous control reference genes has been previously demonstrated (11–15).

Although several miRNA expression profiling studies in pleural effusion specimens have been performed (7–9,16), validation strategies for the selection of reference genes for normalization have, to the best of our knowledge, not been reported. Therefore, the aim of the present study was to identify suitable reference genes for normalizing the expression levels of circulating miRNAs in pleural effusion identified by RT-qPCR.

## Materials and methods

### Patients and pleural effusion samples

Pleural effusion samples were derived from 111 patients who visited Chungbuk National University Hospital (Cheongju, Korea) or Kangwon National University Hospital (Chuncheon, Korea) between February 2009 and September 2012. The pleural effusions were diagnosed as benign, as determined by the clinical context and the absence of malignant cells in at least two separate samples from the same patient. All lung adenocarcinoma-associated MPE (LA-MPE) samples were obtained from patients with tumors that were histologically and clinically diagnosed as primary adenocarcinoma of the lung, and that contained adenocarcinoma cells, as confirmed by pathologists. All samples were transported to the laboratory within 30 min of collection. The samples were then centrifuged at 11,300 × g for 5 min, and the supernatant and sediment fractions were aliquoted into separate microcentrifuge tubes and stored at −80°C prior to use. All patients provided written informed consent, and the study was reviewed and approved by the Institutional Review Board of Chungbuk National University Hospital.

### RNA extraction

RNA was extracted from 500 μl of each sample by the use of a Genolution urine miRNA purification kit (Genolution Pharmaceuticals, Inc., Seoul, Korea), according to the manufacturer’s instructions. The quantity of RNA extracted from each sample was examined using a NanoDrop ND-1000 spectrophotometer (Thermo Scientific, Wilmington, DE, USA).

### Microarray-based miRNA analysis

Candidate reference miRNAs were selected from 10 BPE samples and 10 LA-MPE samples using data from miRNA microarray analyses. The BPE and LA-MPE miRNA expression profiles were generated by hybridizing small RNAs extracted from the pleural effusion samples to locked nucleic acid probes targeting 160 human miRNAs. A PANArray^TM^ miRNA expression profiling kit (Panagene Inc., Daejeon, Korea) was used to identify the candidate reference miRNAs. This kit is a peptide nucleic acid (PNA)-based microarray, which uses a novel miRNA labeling method to examine the expression profiles of cancer- and stem cell-related miRNAs.

### RT-qPCR

The expression levels of the candidate reference miRNAs in 46 BPE samples and 45 LA-MPE samples were validated using RT-qPCR. The expression levels of two small nuclear RNAs (snRNAs; U6 snRNA and RNU6B) and two small nucleolar RNAs (snoRNAs; RNU44 and RNU48) were also measured. Reverse transcription of 100 ng isolated miRNA was conducted using a high-capacity cDNA reverse transcription kit (Applied Biosystems, Foster City, CA, USA), according to the manufacturer’s instructions, and a specific miRNA primer provided with a TaqMan^®^ MicroRNA Assay kit (Applied Biosystems). RT-qPCR was performed using the Applied Biosystems 7,500 Fast Real-Time PCR system along with a TaqMan^®^ MicroRNA Assay, TaqMan^®^ Universal PCR Master mix and No AmpErase^®^ UNG (Applied Biosystems). All reactions were performed in triplicate and Cq data were determined using the default threshold settings. The expression levels of the candidate reference miRNAs and small RNAs included in the final analysis were calculated using the 2^−ΔΔCt^ method.

### Data analysis

Non-parametric tests [Mann-Whitney U test and Kruskal-Wallis one-way analysis of variance (ANOVA) with Dunn’s multiple comparisons correction] were used to determine any statistically significant differences between independent groups. Spearman’s correlation coefficients were used to calculate the associations between the clinical variables and the expression levels of the candidate reference genes. P<0.05 was considered to indicate a statistically significant difference. All statistical analyses were performed using SPSS software for Windows, version 15.0 (SPSS, Inc., Chicago, IL, USA). The NormFinder (http://moma.dk/normfinder-software) and BestKeeper (http://www.gene-quantification.de/bestkeeper.html) software programs were used to analyze the expression stabilities of the reference miRNAs. NormFinder is a Microsoft Excel add-in that uses an ANOVA-based model to calculate stability values from a panel of candidate genes in different subgroups by combining the intra- and inter-group expression variation (17). BestKeeper determines the geometric mean and coefficient of variance (18). Genes with standard deviation >1 were considered to be inconstant. Inter-gene associations were examined by pairwise correlation analysis. This calculation was used to determine whether the genes exhibited similar expression behavior. Candidate reference genes that exhibited high correlation with one another were included in the BestKeeper Index calculation. In the two programs, the lowest stability values indicate the most stably expressed genes. To determine the expression levels of the target miRNAs relative to suitable reference genes, the comparative ΔCt method was used, with the relative quantities (Q_rel_) calculated as follows: Q_rel_ = 2^−Δ(Cq_test sample_ − _Cqnormalizer_)^. When normalized to the sample volume, the relative expression level of a target miRNA was calculated as follows: Q_rel_ = 2^−Δ(_Cqtest sample_ − _Cqaverage of all samples_)^.

## Results

### Selection of candidate miRNA reference genes by microarray analysis

A total of 10 BPE samples and 10 LA-MPE samples were analyzed using miRNA microarrays. The following criteria were used to identify candidate reference miRNAs: miRNAs were detected in all 20 pleural effusion samples, the mean change in miRNA expression levels was <1.1-fold, and no significant differences in miRNA expression levels (P>0.05) between the BPE and LA-MPE samples was detected. To avoid artifacts resulting from normalization of the microarray data, raw microarray expression data were also used. Of the 160 human miRNAs included in the PANArray miRNA expression profiling kit, 59 were identified in all pleural effusion samples. Five of these miRNAs (miR-192, miR-20a, miR-221, miR-222 and miR-16) exhibited mean fold changes of <1.1-fold, with expression levels that did not differ significantly between the BPE and LA-MPE samples.

### Validation of candidate reference genes by RT-qPCR

**Figure 1:**
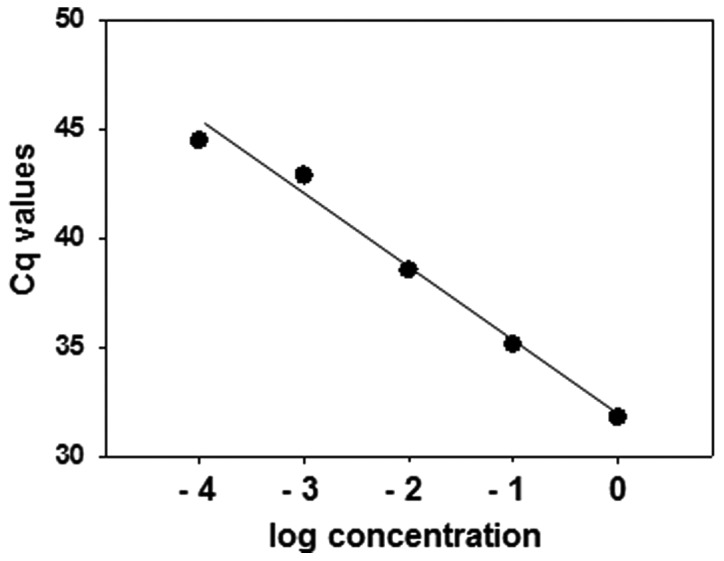
Linear correlation between Cq values and log concentration of miR-192 in a serially diluted pleural effusion sample. miR-192 expression levels were measured in 10-fold serial dilutions of the pleural effusion sample. The concentration of undiluted pleural effusion was arbitrarily designated as 1. Each point represents the mean of duplicate measurements. The regression curve of Cq values versus log concentration reveals a linear correlation (*R*^2^=0.9863) for Cq values between 31 and 44.

**Figure 2:**
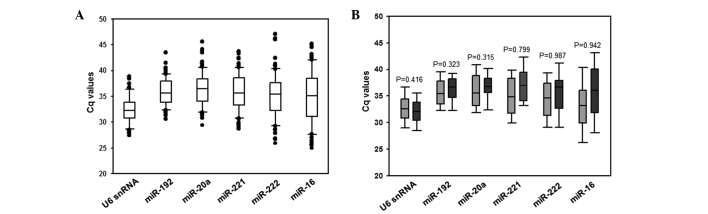
Expression levels of candidate reference genes in benign plural effusion (BPE) and lung adenocarcinoma-associated malignant pleural effusion (LA-MPE) samples. Reverse transcription-quantitative polymerase chain reaction (RT-qPCR) analyses of 46 BPE and 45 LA-MPE samples were performed. (A) Box-and-whisker plot of the primary Cq values of U6 snRNA and five candidate reference miRNAs (miR-192, miR-20a, miR-221, miR-222 and miR-16)in the 91 pleural effusion samples. (B) Box-and-whisker plot of the Cq values of candidate reference genes in the BPE and LA-MPE samples. The expression levels of all candidate reference genes did not differ significantly between BPE and LA-MPE samples (P>0.05). The Cq values were corrected to PCR efficiency and two inter-plate controls. The boxes (light gray, BPE and dark gray, LA-MPE) signify the upper and lower quartiles, the median is indicated by a horizontal line, and the whiskers depict the 10th and 90th percentiles. The P-values of the Mann-Whiney U tests are indicated above each plot.

The baseline characteristics of the patients in the validation cohort are listed in Table I. RT-qPCR was performed to further evaluate the expression patterns of the five candidate reference miRNAs identified by microarray analysis. A cohort of 91 pleural effusion samples, including 46 BPE samples and 45 LA-MPE samples, was used for the RT-qPCR experiments. Furthermore, the set of candidate reference miRNAs was extended by inclusion of the U6 snRNA, RNU6B, RNU44 and RNU48 small RNAs, which are commonly used for miRNA expression level normalization. Details of the functions and PCR amplification efficiencies of each of the candidate reference miRNAs and small RNAs are listed in Table II (19–25). To evaluate the range of detectable Cq values, the miR-192 expression levels in 10-fold serial dilutions of a pleural effusion sample were examined. A plot of Cq values versus the log concentration revealed a linear correlation for Cq values between 31 and 44 (Fig. 1). These results demonstrate that this method of measurement was appropriate for the present study. RNU6B, RNU44 and RNU48 were not detected in the majority of pleural effusion samples; therefore, these small RNAs were excluded from further analysis. The expression levels of the remaining six candidate reference genes, namely miR-192, miR-20a, miR-221, miR-222, miR-16 and U6 snRNA, varied widely across the samples tested, with Cq values ranging between 25.00 and 47.03 (Fig. 2A). The expression levels of all miRNAs did not differ significantly between BPE and LA-MPE samples (Fig. 2B). The expression levels of the five miRNAs and U6 snRNA in BPE and LA-MPE samples did not depend on age (Spearman’s rank correlation ranged between −0.002 and 0.128, P-value ranged from 0.305 to 0.988), gender (Mann-Whitney U test, P-value ranged between 0.279 and 0.808) or diagnosis (Kruskal-Wallis test, P-value ranged between 0.066 and 0.874).

### Stability of the candidate reference gene expression levels

Variations in the expression stabilities of the reference genes were further assessed using the NormFinder and BestKeeper algorithms. The ranking of the genes by these software programs is summarized in Table III; lower stability values indicate higher gene stability. NormFinder identified U6 snRNA as the most stably expressed gene, with a stability value of 0.096. Combining the U6 snRNA and miR-192 gene data further reduced the NormFinder stability value to 0.081. Using the BestKeeper algorithm, a similar ranking order of the candidate reference genes was observed; miR-192 was identified as the most stable gene (stability value of 0.160), followed by U6 snRNA (stability value of 0.177).

### Influence of reference genes on the relative quantification of miR-198

**Figure 3:**
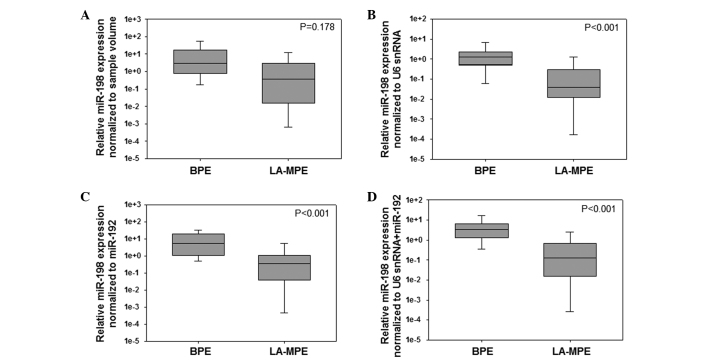
Effects of different normalization methods on the measurement of miR-198 expression levels. (A) No significant differences in the miR-198 expression levels in pleural effusion samples were detected when the data were normalized to serum volume. When the data were normalized to (B) U6 small nuclear RNA expression levels, (C) miR-192 expression levels and (D) a combination of U6 snRNA and miR-192 expression levels, significant differences between benign pleural effusion (BPE) and lung adenocarcinoma-associated malignant pleural effusion (LA-MPE) samples were observed (P<0.001). The boxes signify the upper and lower quartiles, the median is indicated by a horizontal line, and the whiskers depict the 10th and 90th percentiles. The Mann-Whiney U test P-values are indicated in the upper right corner of each plot.

Cell-free miR-198 expression levels have been previously demonstrated to be significantly lower in LA-MPE samples than in BPE samples (9). Therefore, in the present study, to assess the impact of reference gene selection on the validity of miRNA expression data, the effect of normalization to the following variables on the relative quantification of miR-198 expression levels was evaluated: Sample volume; U6 snRNA expression levels (the most stable candidate gene ranked by NormFinder); miR-192 expression levels (the most stable candidate gene ranked by BestKeeper); and the combination of U6 snRNA and miR-192 expression levels. For normalization of the RT-qPCR data to the sample volume, RNA was extracted from 500 μl of each pleural effusion sample and the Cq values were directly converted to the relative quantification values using the formula described above. Using this method, no significant differences in the levels of miR-198 expression were detected between the BPE and the LA-MPE samples (P=0.178; Fig. 3A). However, when data were normalized to the expression levels of U6 snRNA or miR-192, the miR-198 expression levels were significantly lower in the LA-MPE samples than in the BPE samples (P<0.001 for U6 snRNA and miR-192; Fig. 3B and C, respectively). Significant differences in the levels of miR-198 expression between BPE and LA-MPE samples were also detected when the combination of U6 snRNA and miR-192 expression levels served as a normalizer (P<0.001; Fig. 3D).

## Discussion

To the best of our knowledge, the present study is the first systematic investigation of suitable reference genes for the relative quantification of miRNAs in pleural effusion. In the present study, four strategies were used to identify suitable reference genes. miRNA microarray analyses of BPE and MPE samples were performed to identify the invariant miRNAs that are stably expressed. These candidate reference miRNAs, as well as the U6 snRNA, RNU6B, RNU44 and RNU48 small RNAs, which are the most frequently used reference genes in miRNA expression studies (10,11,26), were validated by RT-qPCR. The statistical algorithms NormFinder and BestKeeper were used to identify the most useful endogenous reference genes for relative quantification. The impact of different normalization approaches on the ability to detect reduced miR-198 expression levels in LA-MPE samples was also examined. The results emphasize the importance of an appropriate normalization approach to identify U6 snRNA and miR-192 as suitable reference genes for miRNA analyses in pleural effusion samples.

The selection of appropriate reference genes as normalizers for the relative quantification of miRNA expression levels is required to avoid erroneous results and to improve the comparability of miRNA expression level data amongst studies (10). The conventional approach to miRNA RT-qPCR data analysis is to normalize the values to the expression levels of snRNAs, such as U6 snRNA or RNU6B, or snoRNAs, such as RNU44 or RNU48. Alternatively, synthetic miRNAs, including ath-miR 156a or ath-miR 159a, are commonly added to the samples and used as normalizers. However, these miRNAs may exhibit variable expression levels depending on the samples used and may introduce noise when used as internal controls (11–15). Due to differences in length, the physicochemical properties, isolation efficiencies and degradation rates of small RNAs differ from those of miRNAs (26). In the present study, RNU6B was not detected in the majority of pleural effusion samples; the poor quality of RNU6B as a reference gene has previously been reported in miRNA expression studies of several other types of malignancy (11–14). Unlike miRNAs, RNU6B is a high molecular weight RNA that lacks resistance to degradation by RNase in body fluids (27); these properties may account for the low abundance of RNU6B in the pleural effusion samples included in the present study. Recent studies have also reported that snoRNAs exhibit highly variable levels of expression, and the use of these species as reference genes may introduce noise and result in inaccuracies in the data (15,28). Furthermore, the expression levels of snoRNAs have been associated with cancer prognosis in multiple studies (15,29,30). This evidence demonstrates that these molecules do not meet the requirement that reference genes be unaffected by biological changes, disease or treatments.

Non-human synthetic miRNA also frequently serves as a spike-in control to normalize for technical variability (31). In two recent studies that attempted to identify potential diagnostic or prognostic miRNA biomarkers in pleural effusion, the relative miRNA expression levels were directly normalized using ath-miR156a as a spiked-in reference gene (8,16). However, spike-in controls may not correct for the variability arising from differences in template quality and/or the efficiency of the reverse transcription reaction. Therefore, candidate reference genes must be validated for each individual study design, as even frequently used reference genes are unstable under certain conditions. Thus, the identification of a reference gene that is most suited to the specific samples and experimental conditions of the study is recommended.

In the current study, the candidate reference genes identified by the microarray and subsequent RT-qPCR experiments were analyzed using the NormFinder and BestKeeper algorithms. NormFinder calculated the intra- and inter-group variations and identified U6 snRNA as the single most stable reference gene with a stability value of 0.096. The use of more than one reference gene is generally considered to increase the accuracy of miRNA quantification, and the combination of U6 snRNA and miR-192 expression exhibited an improved NormFinder stability value of 0.081. NormFinder and BestKeeper did not produce the same reference gene ranking order for normalization; this difference may be attributable to the different calculation models used in the tools. However, NormFinder and BestKeeper ranked U6 snRNA and miR-192 as the top two reference genes.

The impact of different normalization strategies on the accuracy of RT-qPCR results was examined by analyzing the expression levels of cell-free miR-198 in pleural effusions. In a previous study, the levels of miR-198 expression were found to be significantly lower in LA-MPE samples than in BPE samples, indicating that this miRNA may have diagnostic potential for differentiating BPE and MPE (9). In the present study, the relative quantification of miR-198 was assessed using different normalization strategies. When the miR-198 data were normalized to the sample volume, no significant differences between BPE and LA-MPE samples were detected. However, when the data were normalized to U6 snRNA expression levels, miR-192 expression levels, and a combination of U6 snRNA and miR-192 expression levels, significant differences between BPE and LA-MPE samples were detected. These results indicate that normalization of miRNA expression level data to those of systematically selected reference genes detects smaller differences than those identified by normalization of the data to the sample volume.

The present study has certain limitations. A relatively small number of well-known miRNAs (160 of the >1,000 currently available) were screened using the PNA-based microarray. Furthermore, the number of candidate reference genes examined was small; the use of multiple reference genes is critical to achieve more reliable expression data (32). Another limitation of the study was that the identification of U6 snRNA and miR-192 as reference genes was only confirmed by normalization of the expression levels of a single miRNA (miR-198) in the pleural effusion samples. If further studies investigating miRNAs as potential diagnostic, prognostic and predictive biomarkers in pleural effusion are to be undertaken, the use of U6 snRNA and miR-192 as reference genes must be further validated. Another issue is that all MPE samples used in the present study were obtained from lung adenocarcinoma specimens; whether U6 snRNA and miR-192 may be used as reference genes for other types of MPE remains to be determined.

In conclusion, the use of the expression levels of U6 snRNA, miR-192 or a combination of these genes for the relative quantification of miRNA expression levels in pleural effusion is recommended, as determined by the results from the present study.

## Figures and Tables

**Table I:** Baseline characteristics of the patients.

Clinical characteristics	BPE, n (%) (n=46)	LA-MPE, n(%) (n=45)
Age (years)^a^	63 (22–92)	68 (33–92)
Gender		
Male	31 (67.4)	23 (51.1)
Female	15 (32.6)	22 (49.9)
Diagnosis		
Tuberculosis	22 (47.8)	-
Pneumonia	19 (41.3)	-
Transudate	5 (10.9)	-
Metastatic sites		
M1a	-	18 (40.0)
M1b	-	27 (60.0)

a Median (range).

BPE, benign pleural effusion; LA-MPE, lung adenocarcinoma-associated malignant pleural effusion; M1a, separate tumor nodule(s) in a contralateral lobe, a tumor with pleural nodules or malignant pleural (or pericardial) effusion; M1b, distant metastasis.

**Table II:** Details of candidate reference genes.

Gene	RNA species	Accession number	Function	Reference	PCR efficiency (%)
miR-192	miRNA	MI0000234	Regulates dihydrofolate reductase and cellular proliferation	19	98.8
miR-20a	miRNA	MI0000076	Negatively regulates autophagy	20	99.5
miR-221	miRNA	MI0000298	Regulates cell cycle	21	100.9
miR-222	miRNA	MI0000299	Regulates cell cycle	21	101.1
miR-16	miRNA	MI0000070	Induces apoptosis by targeting BCL2	22	98.2
U6 snRNA	snRNA	NR_003027	Guides post-transcriptional modification of cellular RNAs	23	100.0
RNU6B	snRNA	NR_002752	Guides post-transcriptional modification of cellular RNAs	23	95.5
RNU44	snoRNA	NR_002750	Guides site-specific ribosomal RNA modification	24	Undetected
RNU48	snoRNA	NR_002745	Guides methylation of 28S ribosomal RNA	25	Undetected

PCR, polymerase chain reaction; miRNA, microRNA; snRNA, small nuclear RNA; snoRNA, small nucleolar RNA; BCL2, B-cell lymphoma 2.

**Table III:** Ranking and best combination of candidate reference genes in pleural effusion samples as determined by the expression stability values calculated using NormFinder and BestKeeper.

	NormFinder		BestKeeper	
		
Rank	Gene	Stability value^a^	Gene	Stability value^a^
1	U6 snRNA	0.096	miR-192	0.160
2	miR-192	0.103	U6 snRNA	0.177
3	miR-20a	0.158	miR-20a	0.219
4	miR-221	0.185	miR-222	0.222
5	miR-222	0.198	miR-221	0.267
6	miR-16	0.251	miR-16	0.374
Best combination	U6 snRNA/miR-192	0.081	-	-

a High expression stability is indicated by a low stability value.

snRNA, small nuclear RNA; miRNA, microRNA.
